# Oxidative stress: The nexus of obesity and cognitive dysfunction in diabetes

**DOI:** 10.3389/fendo.2023.1134025

**Published:** 2023-04-03

**Authors:** Huimin Li, Jing Ren, Yusi Li, Qian Wu, Junping Wei

**Affiliations:** ^1^ Guang’anmen Hospital, China Academy of Chinese Medical Sciences, Beijing, China; ^2^ Graduate School of Beijing University of Chinese Medicine, Beijing, China

**Keywords:** oxidative stress, obesity, cognitive dysfunction in diabetes, reactive oxygen species, insulin resistance, neuroinflammation, lipid metabolism disorders

## Abstract

Obesity has been associated with oxidative stress. Obese patients are at increased risk for diabetic cognitive dysfunction, indicating a pathological link between obesity, oxidative stress, and diabetic cognitive dysfunction. Obesity can induce the biological process of oxidative stress by disrupting the adipose microenvironment (adipocytes, macrophages), mediating low-grade chronic inflammation, and mitochondrial dysfunction (mitochondrial division, fusion). Furthermore, oxidative stress can be implicated in insulin resistance, inflammation in neural tissues, and lipid metabolism disorders, affecting cognitive dysfunction in diabetics.

## 1 Introduction

The prevalence of obesity has been on the rise globally for the last half century (1). Obesity prevalence has doubled since 1980 in more than 70 countries. Furthermore, women of all ages had a higher prevalence of obesity than men (2). Obesity causes many twenty-first-century chronic diseases worldwide and imposes enormous socioeconomic burdens (1). Numerous risk factors for chronic diseases, including cardiovascular disease (CVD) (3), type 2 diabetes (T2DM), and cognitive impairment, are influenced by obesity (4). Diabetes prevalence has been increasing, especially with T2DM, due to changes in lifestyle factors such as diet, obesity, and lack of exercise (5). The IDF Diabetes Atlas indicates that prevalence in 20–79-year-olds in 2021 was estimated to be 10.5% (536.6 million people), rising to 12.2% (783.2 million) in 2045 (6). Patients with diabetes are also at risk for complications as they age (7). Diabetes patients have been found to have an increased risk for dementia (8). In a US study, people with diabetes had an overall prevalence of dementia and cognitive impairment of 13.1% for people aged 65–74 and 24.2% for those aged 75 and over (9). Those who suffer from cognitive impairment in diabetes experience cognitive dysfunction, delayed executive, function, and impeded information processing speed, and pathology may include neuro amyloid plaques and tau protein tangles (10). There is a correlation between diabetes and cognitive impairment, which negatively affects patient quality of life (11). A cross-sectional analysis of baseline data shows that high BMI and low mood are associated with worse cognitive function among overweight/obese elderly with metabolic syndrome (12). In older people, BMI has been associated with a higher risk of developing type 2 diabetes (13). This review aims to explore how oxidative stress processes could contribute to obesity-related cognitive dysfunction in diabetics.

Oxidative stress (OS) regulates biological components, and it has been proposed to be a mediator of the relationship between obesity and cognitive impairment in diabetes. In 1985, “oxidative stress” was introduced as a concept in redox biology and medicine (14); the concept of biological oxidative stress was defined as “an imbalance between oxidants and antioxidants in favor of oxidants, leading to a disruption of redox signaling and control and molecular damage” (15). Redox reactions contribute to regulation, where endogenous and exogenous regulatory factors, such as the number of biochemical components (oxygen, nitrogen, and sulfur), can contribute to the oxidative stress. Furthermore, these reactive species, called reactive substances, mainly reactive oxygen species (ROS), reactive nitrogen species (RNS), and reactive sulfur species (RSS), stimulate the metabolic processes of cells (16). Reactive species participate in several oxidative signaling pathways, such as NF-κB, JAK-STAT, Nrf-2, and HIF-1; they are also involved in the development of several diseases, including cardiovascular diseases, cancer, and diabetes (17). The production of ROS contributes to the inflammatory response process, which leads to an increase in adipocyte size, promotes adipogenesis and lipogenesis, and adipocyte differentiation (18). Studies have demonstrated that fat accumulation is an early trigger and a fundamental cause of obesity-associated metabolic syndrome resulting in increased oxidative stress (19). Prolonged exposure of adipocytes to ROS leads to insulin-induced activation of PI3-kinase and Akt, resulting in impaired islet function and facilitated glucose transporter member 4 (GLUT4) translocation(20). Due to their sensitivity to oxidative damage, neuronal cells are especially susceptible to neurodegenerative diseases, such as diabetes-related cognitive impairment (21). Mitochondrial homeostasis plays a crucial role in maintaining neuronal and axonal energetic homeostasis. Bioenergetic deficits contribute significantly to the cognitive decline observed in aging and neurodegenerative diseases. Neurons are particularly susceptible to mitochondrial dysfunction due to their intrinsic properties (22). ROS synthesis is derived from mitochondria, and when mitochondria become dysfunctional, ROS production of ROS and oxidative stress increase, and mitochondrial maldistribution disrupts neuronal axonal energy homeostasis. Oxidative stress disrupts neurological metabolism resulting in hypoglucose metabolism in the brain (23). The brains exhibit structural changes due to an accumulation of disease-specific protein aggregates (24). These structural changes may contribute to neuronal and synaptic dysfunction, resulting in cognitive impairment (25).

## 2 The link between obesity and oxidative stress

Oxidative stress is produced by reactive oxygen/nitrogen species (ROS/RNS) (26). Furthermore, oxidative stress alters the balance between the production of ROS and antioxidant defenses. By-products of aerobic metabolism, ROS, can pose a health risk when exposed to stressful environments (27). ROS are primarily derived from mitochondria and electron transport chain (ETC), in which mitochondria produce adenosine triphosphate (ATP) through a series of oxidative phosphorylation processes. However, ROS also contain a variety of chemical entities, including nitric oxide, peroxynitrite, hypochlorous acid, singlet oxygen and hydroxyl radicals (28).

**Figure 1:**
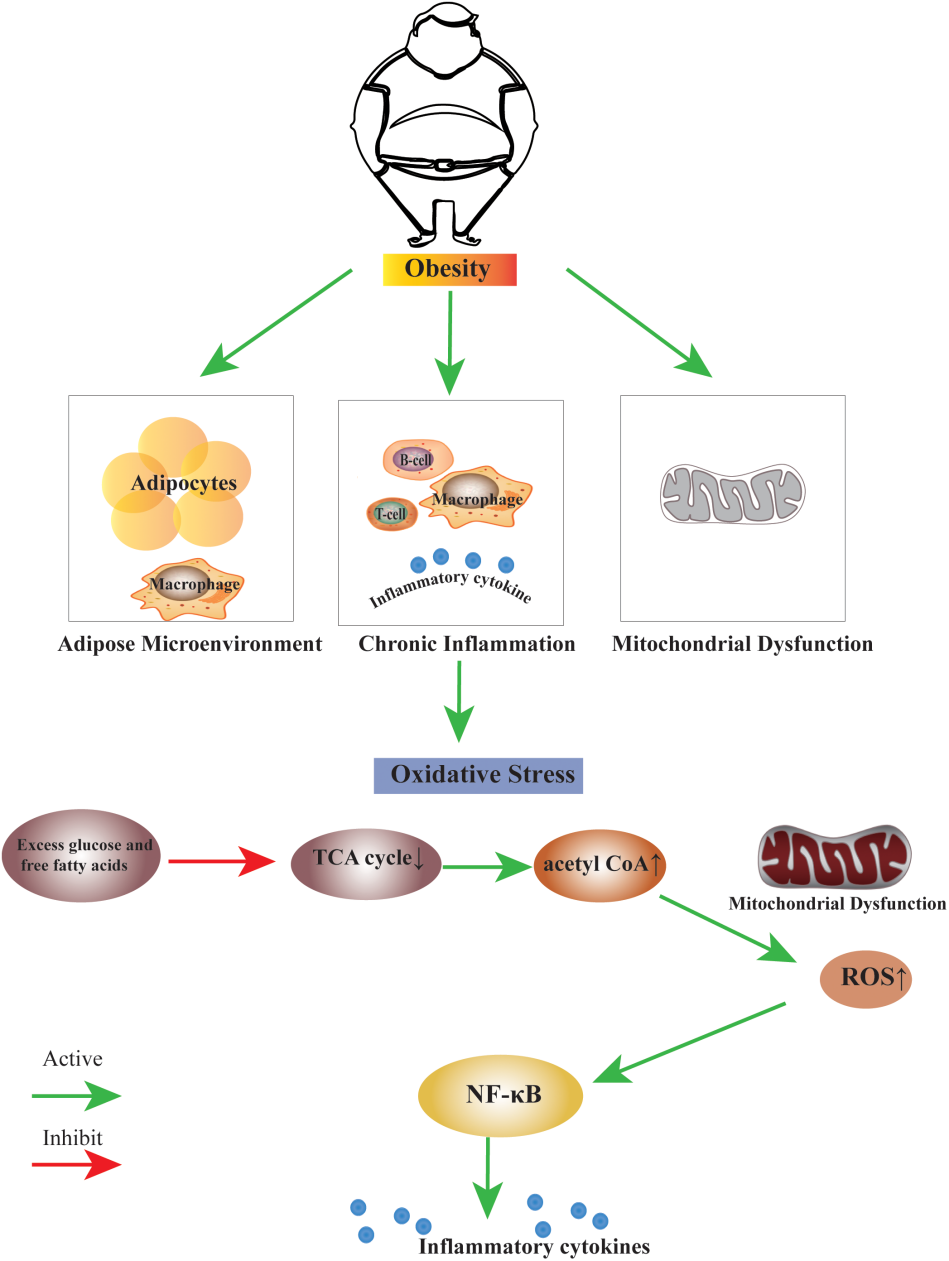
The link between obesity and oxidative stress. Obesity can induce the biological process of oxidative stress by disrupting the adipose microenvironment, mediating chronic inflammation, and mitochondrial dysfunction. The process is probably that excess glucose and free fatty acids suppress the TCA cycle, leading to an increase in the production of acetyl CoA. Excess acetyl CoA stimulates mitochondrial dysfunction, resulting in an increase of ROS within the cell, this change may activate many factors, the nuclear factor κB is the main inflammatory factor.TCA cycle, Tricarboxylic Acid cycle; acetyl CoA, Acetoacetyl coenzyme A; ROS, Reactive Oxygen Species; NF-κB, nuclear factor κB.

Several studies have shown that obesity induces the formation of oxidative stress. The high-fat diet induces oxidative stress in the white adipose tissue of rats (29). When there is a high intake of nutrients, oxidative stress increases, and inflammation is induced through signaling pathways mediated by the nuclear factor-kappa B (30). High consumption of fat-rich diets promotes mitochondrial β-oxidation of free fatty acids (FFAs), and subsequent use of cytochrome-c oxidase leading to excess electron flow increases the accumulation of ROS, ROS, and lipid peroxidation deplete vitamins and antioxidant enzymes (31). We summarise the link between obesity and oxidative stress in terms of disruption of the adipose microenvironment, chronic inflammation in obesity, and mitochondrial dysfunction (**Figure 1**).

### 2.1 Disruption of the adipose microenvironment

Obesity is an increase in lipid content in adipose tissue, manifested by an increase in the size and number of adipose cells (32). Adipose tissue can be divided into three categories: white adipose tissue (WAT), brown adipose tissue (BAT) and beige adipocytes.

White adipocytes are the primary cell type found in human adipose tissue. Energy-yielding triglycerides and cholesterol esters are stored within the sizeable intracellular lipid droplets. Leptin, adiponectin, and other adipokines are among the proteins secreted by white adipocytes.

Brown adipocytes: BAT is widely present throughout the body (33); BAT is rich in multiple lipid droplets and contains uncoupling protein 1– containing mitochondria; these adipocytes mediate thermogenic respiration (34).

Beige adipocytes: Beige adipocytes are derived from white adipocytes tissue, and browning of white adipocytes tissue can be induced by cold stimulation, exercise, and some endocrine hormones; beige adipocytes have thermogenic effects because of rich uncoupling protein 1 (35).

The different adipocytes and macrophages of the adipose tissue constitute the adipose microenvironment. Dysfunction at the WAT level may influence the development of obesity-associated metabolic complications (36). An obesity-induced immune response occurs when metabolic cells (including adipocytes) are involved (e.g., adipocytes). Overnutrition leads to adipotoxicity, which produces inflammatory factors (37). Studies have shown that macrophages infiltrate adipocytes in obese individuals and promote an inflammatory response (38). Kinase inhibitors (IKK), c-jun n-terminal kinase (JNK), and protein kinase r (PKR) can transmit nutrient signals from metabolic tissues to inflammatory cells. This process is accompanied by oxidative stress, and these kinases and their downstream pro-inflammatory targeting factors can be significantly upregulated in obese subjects (39). Due to the accumulation of oxidative biomolecules in adipocytes, the homeostatic system that regulates oxidative stress and the antioxidant regulatory system are suppressed mainly in obese adipocytes. Excess ROS irreversibly damages DNA, lipids, and proteins and adversely affects cellular function (28).

### 2.2 Chronic inflammation in obesity

Obesity is primarily caused by an energy imbalance between excessive calories consumed and insufficient calories expended (40). Adipose tissue is regarded as an energy storage for calories and an essential endocrine organ. It produces many bioactive molecules, including chemokines and cytokines, called adipokines (or adipocytokines), when secreted by adipose tissue. They are not only regulators of systemic metabolism, but also have immunomodulatory properties (41). Adipose tissue is responsible for the production and secretion of many biologically active adipokines, including leptin, adiponectin, resistin, visfatin, and schelatin, that can lead to chronic complications (42). Obesity leads to an increase in adipocytes and enlargement of adipose tissue. The ensuing decrease in oxygen tension leads to hypoxia and massive accumulation of hypoxia-inducible factor (HIF-1) in adipocytes. Furthermore, hypoxia has been linked to adipose inflammation and macrophage infiltration (43). Studies have shown that macrophages accumulate in adipose tissue of obese people as well as in the obese B6.V Lepob/ob mouse model, and macrophages promote the secretion and expression of adipokines, including tumor necrosis factor-alpha (TNF-α), iNOS and interleukin-6 (IL-6) (38).

Obesity is a chronic low-grade inflammatory condition, with adipose tissue infiltrated by macrophages and elevated inflammatory markers and cytokines. This low-grade chronic inflammation in adipose tissue may contribute to developing related metabolic diseases, such as insulin resistance and T2DM (44). Adipocytes produce large amounts of adipokines with inflammatory functions, such as IL-6, IL-1, and TNF-α, which induce ROS production and mediate oxidative stress (45). TNF-α is produced mainly by macrophages and is also a critical adipokine. Fat accumulation leads to adipocyte damage, leading to high production of cytokines such as TNF-α, which produces ROS in tissues and increases the rate of lipid peroxidation (46). TNF-α also activates the NF-kB signaling pathway to aggravate the inflammatory response (47). During oxidative stress, adipokines, including leptin, IL-6, and lipocalin, resist all functions (45).

Oxidative stress impairs islet beta-cell function in several ways; it significantly reduces insulin production, impairs the ability of insulinogenic vesicles to enter the plasma membrane, and reduces the response to hyperglycemia. Oxidative stress can induce islet β-cell apoptosis, and excess free radicals interfere with β-cell neogenesis (48). Oxidative stress leads to reduced GLUT4 expression and ultimately reduces insulin sensitivity by disrupting the binding of nuclear proteins to the insulin response element in the GLUT4 promoter (49). Oxidative stress was also involved in the development of diabetic encephalopathy. Oxidative stress inhibits the islet signaling system. HFD/STZ induced a significant increase in relevant oxidative stress parameters such as TBARS, NO levels, and XO activity in the brain tissue of rats compared with controls, and serum peripheral TNF-α and IL-6 inflammatory cytokine levels were significantly increased in the diabetic rats, and a similar brain AD-related miRNA expression profile was observed in the diabetic rats (50).

### 2.3 Mitochondrial dysfunction

Mitochondria are intracellular organelles that play an important role in the cell by metabolizing nutrients and producing adenosine triphosphate (ATP). Mitochondria regulate energy, maintenance of cellular calcium homeostasis, production and removal of reactive oxygen species, and regulation of cell death (51). Mitochondria produce energy in the form of ATP through the oxidative metabolism of nutrients, consisting of two main steps: 1) oxidation of NADH or FADH2 produced during glycolysis, TCA or β-oxidation of fatty acids, with most of the ATP being produced through the TCA cycle through the ETC; 2) oxidative phosphorylation (OXPHOS) to produce ATP. Mitochondria continuously metabolize oxygen and produce ROS during the combination of electron transport and protons in the ETC, which is the primary source of ROS (52).

Mitochondrial dysfunction can manifest itself by loss of mitochondrial membrane potential, altered ETC function, increased ROS production, and decreased oxygen consumption. There is a reduction in the efficiency of mitochondrial ATP production (53). Mitochondrial dysfunction can also occur when mitochondrial molecular dynamics is impaired. Mitochondria is a dynamic energy organelle that responds to energy demands and environmental stimuli through fusion, fission, and movement to maintain cellular homeostasis (54). Fission can also promote mitochondrial autophagy and biogenesis, two events that can occur because of mitochondrial fission (55, 56). Mitochondria generate several stress response pathways, including the mitochondrial unfolded protein response and degrading mislocalized proteins in mitochondria dysfunction (57, 58). Severely damaged mitochondria can be identified and degraded through the process of mitochondrial autophagy (59). When mitochondrial autophagy is dysregulated, ROS produced by mitochondria can activate inflammatory vesicles composed of NLRP3, the bridging protein ASC, and caspase-1, triggering inflammation. It has been reported that defects in the autophagy gene PINK1 increase NLRP3 expression and lead to brown fat dysfunction in mice (60, 61).

Studies have shown that excessive nutrient intake leads to hyperglycemia, increases ROS production, and causes mitochondrial dysfunction in adipocytes, suggesting that obesity triggers oxidative stress and mitochondrial dysfunction (57). When mitochondrial function is impaired, major adipocyte pathways are altered, resulting in decreased adipogenesis, increased lipolysis, and decreased fatty acid esterification; these alterations promote changes in insulin sensitivity (62). A study showed that high fat-induced obese (DIO) mice exhibit insulin resistance, mitochondrial dysfunction, hepatic lipid deposition, and oxidative stress (63). A study indicated that the expression of mitofusin-2 (Mfn2, a mitochondrial fusion protein) was decreased in the muscles of obese subjects or type 2 diabetics, leading to an imbalance between mitochondrial fusion and fission events and mitochondrial dysfunction, which may be involved in insulin resistance (64). Significantly elevated levels of acylcarnitine in patients with nonalcoholic fatty liver mark mitochondrial dysfunction and impaired fatty acid oxidation (65). A study indicated a decrease in mitochondrial biosynthesis in a rodent model of obesity (66). Down-regulation of mitochondrial biogenesis in obesity is associated with metabolic alterations, insulin resistance, and low-grade inflammation (67). Chronic high-fat diet feeding promotes excessive apoptosis in mouse HK-2 cells by inducing oxidative stress and mitochondrial disorders in kidney cells (68). It has been shown that a high-fat diet induces oxidative damage in the brain of obese (DIO) rats and that a high-fat diet increases lipid oxidation in the brain tissue of DIO rats as well as the level of mitochondrial ROS (69). Mitochondrial dysfunction in the brain due to obesity may lead to insulin resistance and cognitive dysfunction(70, 71). Jheng found smaller and shorter mitochondria and increased mitochondrial fission in the skeletal muscle of obese mice, suggesting that altered mitochondrial fission is associated with mitochondrial dysfunction in the skeletal muscle and insulin resistance (72).

A high-fat diet induces mitochondrial expansion in rodent brown fat, and excess leads to inhibition of mitochondrial fusion, resulting in fragmentation and autophagy, leading to mitochondrial dysfunction (73). The excess leads to cellular oxidative stress, which subsequently induces an inflammatory cascade response. This process is likely caused by excess glucose and free fatty acids suppressing the TCA cycle, increasing acetyl CoA. Excess acetyl CoA stimulates the mitochondria to produce excess superoxide in the electron transport chain, which increases ROS within the cell. This change may activate many factors; the nuclear factor κB is the primary inflammatory factor (74).

## 3 Oxidative stress and cognitive dysfunction in diabetes

**Figure 2:**
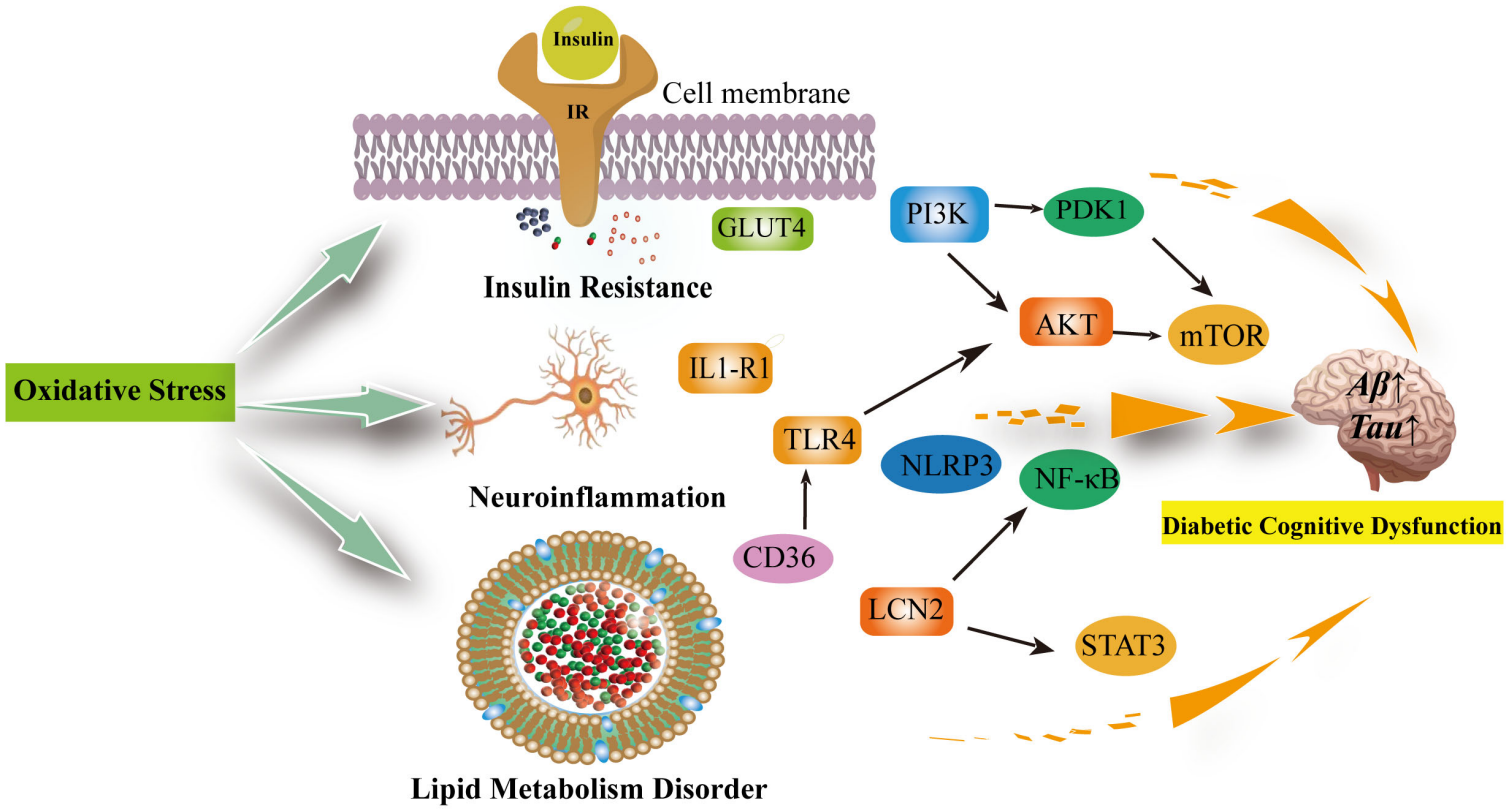
Oxidative stress can be involved in insulin resistance, neuroinflammation, lipid metabolism disorders leading to diabetic cognitive dysfunction. Oxidative stress reduces GLUT-4 expression and the translocation of GLUT-4 to the cell membrane, decreasing insulin sensitivity; In the brain, insulin activates the PI3K/PDK1/AKT and the PI3K/AKT/mTOR signaling pathway to inhibit apoptosis and promote neuronal development and survival, which are inhibited when insulin is resistant and increase the production of inflammatory factors. Neuroinflammation is associated with excessive microglia activation. activation of IL1-R1 signaling pathway, NLRP3/IL-1β signaling pathway, NK-κB signaling pathway and release of pro-inflammatory factors exacerbate neuroinflammation and neuronal damage in the brain. The inhibition of TLR4/AKT/mTOR signaling pathway inhibits cellular autophagy as well as promotes neuroinflammation and microglia apoptosis. Diabetic cognitive dysfunction is also exacerbated by the presence of impaired lipid metabolism in the brain. CD36 recognizes oxidized low-density lipoprotein receptors (TLRS) and triggers a toll-like response to stimulate sterile inflammation. Meanwhile, LCN2 is mainly produced in glial cells of the brain under oxidative stress. It promotes cellular neuroinflammation by activating the NF-kB pathway as well as the STAT3 signalling pathway to promote microglia activation. IR, Insluin resistance; GLUT4, facilitated glucose transporter member 4; PI3K, phosphatidylinositol 3' -kinase; PDK1, pyruvate dehydrogenase kinase isoform 1; AKT,  Protein Kinase B; mTOR, mammalian target of rapamycin; TLR4, toll-like receptor 4; IL1-R1, interleukin 1 receptor type I; NLRP3,NLR family pyrin domain containing 3; NF-κB, Nuclear factor kappa B; LCN2, Lipocalin 2; STAT3, signal transduction and transcription 3; CD36, Platelet glycoprotein 4.

Oxidative stress is an imbalance between the production of oxidants and antioxidant defenses that may result in damage to biological systems. Oxidative stress is considered one of the crucial factors in the development and progression of cognitive impairment in diabetes mellitus (75). In cognitive impairment, there are interconnections between oxidative stress, insulin resistance, neuroinflammation, and abnormal lipid metabolism (76–78) (**Figure 2**).

### 3.1 Oxidative stress and insulin resistance in diabetic cognitive dysfunction

Insulin resistance means that systemic target tissues such as fat, muscle, and liver are less sensitive to insulin and cannot properly regulate the pathological state of glucose homeostasis (79). Inflammation, dysfunction, and elevated OS levels lead to insulin signaling cascade disorder and are important triggers of insulin resistance (80–82). In pro-inflammatory conditions, activation of glial cells can lead to progressive neuronal damage (83). Additionally, insulin regulates metabolic pathways that maintain learning and memory at the brain level and glucose transport/metabolism (83). In diabetic cognitive dysfunction, insulin resistance weakens the metabolic raw material of dysfunctional neurons and affects memory function (84).Currently, the molecular mechanism of the development of insulin resistance has not been fully elucidated. Insulin receptor substrates (IRSs) work as scaffold protein driving activation of two primary insulin signaling pathways: 1) PI3K/PDK1/Akt pathway; And 2) MAPK pathway (85). The former is closely related to insulin metabolism, while the latter is mainly involved in cell growth differentiation (86).

ROS can act as both a signaling agent and a damaging agent. Low levels of endogenous reactive oxygen species play an essential role in the signaling pathway and have crucial physiological significance (87). In insulin signaling, there are redox initiation steps in which some oxidizing agents, such as hydrogen peroxide (H_2_O_2_), promote the phosphorylation of insulin receptors (83). In addition, it can inhibit protein tyrosine phosphatase PTP1B, which deactivates IR by dephosphorylating A-ring phosphotyrosine (88).Thus, insulin-induced H_2_O_2_ acts as a net positive regulator in acting insulin receptors. Furthermore, as age increases, OS levels increase and glutathione (GSH) levels decrease, which has been verified in aging models (89). In insulin resistance or T2DM mice, oxidative stress markers increased, and glutathione levels decreased in the brain(90). Previous studies have shown that brain plasticity, the ability of the brain to undergo structural and functional changes due to environmental stimulation, is carefully regulated by dietary and nutritional-dependent hormones, including insulin (91). Therefore, it can be shown that OS is closely related to the development of insulin resistance and cognitive dysfunction. Changes in insulin signals in the central nervous system can accelerate brain aging, affect brain plasticity, and promote synaptic loss and nerve degradation (92).

Oxidative stress can cause β-cell dysfunction (93). Because the antioxidant defense system of β-cell is low, OS is widely found in diabetes mellitus and plays an essential role in β-cell dysfunction (48, 93). OS can reduce the production of insulin, impair the contents of the original insulin vesicles into plasma membrane, and reduce the exocytosis of glucose into circulation (93, 94). Since apoptotic agents are highly sensitive to OS, OS can induce pancreatic cell apoptosis and lead to β-cell apoptosis (94, 95). An overload of free radicals can affect the normal metabolic pathway in β-cells, damage the K_ATP_ channel, and lead to decreased insulin secretion (93, 96). Previous studies have shown that OS activates Nf-*κ* B, JNK/SAPK, p38 MAPK, hexosamine pathway, and toll-like receptor (TLRs), thereby impairing β-cell function (48, 93). In addition, β-cell mitochondrial dysfunction induced by oxidative stress may be an important mechanism leading to β-cell dysfunction (48, 93). β-cell dysfunction, which results from oxidative stress, can lead to insulin resistance and in turn diabetes cognitive dysfunction.

Oxidative stress can reduce insulin sensitivity in insulin-dependent cells such as adipocytes and myocytes (48). Normal GLUT-4 expression and localization are necessary to maintain these tissues’ insulin sensitivity (97). Reduction of GLUT-4 expression/localization is one of the main molecular mechanisms by which oxidative stress induces insulin resistance and promotes the development of cognitive dysfunction in diabetes mellitus (48). Studies have found lower expression and localization of GLUT-4 in patients with insulin resistance and T2DM (98–100). Oxidative stress can reduce the translocation of GLUT-4 to the cell membrane (101). Long-term oxidative stress inhibits transcription factors and microscopic RNAs involved in GLUT-4 expression (101–103).

Oxidative stress can impair normal insulin signal transduction (IST) at different levels (104). Hyperglycemic-induced OS activates different stress-sensitive serine/threonine (Ser/Thr) kinases such as IKK-*β*, which phosphorylates multiple targets such as IR, IRS-1 and IRS-2, leading to adverse downstream effects, including decreased PI3K activation and insulin resistance (48, 105). Oxidative stress can damage insulin sensitivity and lead to insulin resistance and cognitive dysfunction of diabetes by downregulating the proteins involved in normal IST, such as Insulin-degrading enzyme (IDE), Biliverdin reductase-A (BVR-A), Akt, IRS, IRS-1 and GSK-3 (48, 83, 50). Therefore, IST abnormality is one of the important mechanisms of insulin resistance caused by oxidative stress.

Insulin resistance can occur in both obesity and diabetes and is manifested in peripheral and central insulin resistance (106). In the brain, insulin acts as a neuromodulator to regulate activity-dependent synaptic plasticity by activating PI3K/PDK1/Akt signaling pathways (107). Insulin can inhibit apoptosis by activating the Akt signaling pathway to promote neuronal cell survival (108). Insulin resistance is characterized by the down-regulation of insulin receptor expression and impaired IRS proteins (86). IRs are localized in both presynaptic and postsynaptic neurons (109, 110). IRs recruit and activate PI3K complexes, which subsequently activate AKT (111), AKT downstream of GLUT4 and mTOR complexes, AKT-mediated stimulation of mTOR and its downstream targets regulates protein and lipid synthesis and promotes dendritic spine formation, as well as many aspects of neuronal development, survival, autophagy, and long-term synaptic plasticity (84, 111). When oxidative stress processes activate kinases such as JNK and IKK in neurons, insulin signaling pathways become abnormal, such as the P13K/AKT pathway, thereby the downstream of the pathway is inhibited (112). Failure of insulin signaling causes tau protein hyperphosphorylation (113), in addition to increased neurotoxic Aβ deposition at specific levels of hyperinsulinemia (114), all of which can lead to decreased cognitive function in diabetes. In addition, chronic elevated blood glucose can induce inflammation and can cause insulin resistance. Pro-inflammatory mediators such as TNF-α, IL1-β and IL-6 further exacerbate the inflammatory state through feedback inhibition of insulin receptors and through feedforward mechanisms that disrupt mitochondrial function to stimulate the production of reactive oxygen species, thereby producing an inflammatory environment with reduced insulin sensitivity. This chronic inflammatory environment increases NF-κB inducible kinase (NIK), which independently impairs mitochondrial function to further promote insulin resistance (86). Pro-inflammatory cytokines secreted into the bloodstream (across the blood-brain barrier) during chronic hyperglycemia and inflammatory cytokines within the brain’s innate immune system, soluble misfolded Aβ can induce inflammatory cytokines (e.g., TNF-α) through a NIK-dependent pathway that can lead to neuroinflammation and exacerbate cognitive deficits (115).

### 3.2 Oxidative stress and neuroinflammation in diabetic cognitive dysfunction

Neuroinflammation is the inflammatory response of factors of the central nervous system (CNS) acting on homeostasis in the body (116). This response includes distinct types of cells in the central nervous system, such as microglia and astrocytes (116, 117). Neuroinflammation aims to restore neuronal homeostasis and protect neuronal integrity (118). During the acute phase, neuroinflammation can protect neurological homeostasis, promote nerve growth, repair damaged cells, and remove protein plaques (119). During the chronic phase, neuroinflammatory-induced maladaptive results will cause neuronal damage to worsen (119). Neuroinflammation is a common pathogenic factor in neurological disorders, including Alzheimer’s disease (AD), diabetic cognitive dysfunction, and depression (120). Neuroinflammation includes various inflammatory events in the central nervous system under pathological conditions. Brain alterations in AD and diabetic cognitive dysfunction can manifest amyloid-β plaques and are associated with neuroinflammation (112). Abnormal activation of glial cells (microglia and astrocytes) can mediate neuroinflammation leading to neurodegenerative disease, and dysfunctional neurons alter the clearance of amyloid-β plaques in AD, promoting neuroinflammation and cognitive impairment (121). Obesity-induced chronic inflammation also affects the central nervous system (122), with obesity-promoting peripheral inflammation and increasing the permeability of the blood-brain barrier (BBB), and elevated levels of inflammatory mediators in diabetic patients promote neuroinflammation by triggering harmful neutrophil/microglia activation in the diabetic brain (123). Elevated levels of related proteins in inflammatory cells (e.g., lipid transport protein 2, LCN2 and tension enhancer binding protein, TonEBP) can adversely affect diabetic encephalopathy by leaking through the BBB (124).

Neuroinflammation and oxidative stress are essential in the onset and development of neurodegenerative lesions and are closely linked in their pathogenesis. ROS and RNS can further enhance intracellular signaling cascades and increase the expression of pro-inflammatory factors. On the other hand, inflammatory cells secrete active substances that produce ROS (116, 125). Therefore, neuroinflammation and oxidative stress can stimulate and interact with each other. An imbalance in redox and insufficient inflammatory response in the central nervous system causes neuroinflammation (116).

The blood-brain barrier (BBB) is a protective barrier for the CNS that prevents harmful substances from entering the brain and maintains intracerebral homeostasis by regulating the transport of essential molecules, including glucose. Pericytes and astrocytes contribute to the formation of the basal membrane of the blood-brain barrier (126, 127). Hyperglycemia increases the rate at which pericytes and astrocytes respire, producing ROS production and oxidative stress in people with diabetes (127, 128). Increased ROS further stimulates the upregulation of inflammatory cytokines and activates the NF-*κ*B signaling pathway, leading to leakage of the blood-brain barrier (129). Neuroinflammation resulting from these injuries promotes the opening of the BBB and the influx of high blood sugar into the CNS (127). Long-term high glucose levels can cause disturbances in glucose metabolism pathways, decrease essential cofactors in redox reactions, including NADPH and NAD +, and produce advanced glycosylated end products (AGEs) (130–132). AGEs bind to AGE receptors (RAGEs) on the cell surface, producing excessive ROS (127, 133). High concentrations of ROS can initiate misfolding of proteins in neurons mitochondria, causing dysfunction of mitochondria, causing neuroinflammation, exacerbating tissue damage, and destroying neuronal regeneration (127, 134).

Mitochondria generate the energy required for almost all biological functions of the body and are an essential organelles. Neurons have high energy requirements, and neuronal mitochondria provide constant energy to neuronal cells (127, 135). Mitochondrial dysfunction causes intracellular energy, leading to inflammation and cell death (119). Previous studies have found that brain neurons are more susceptible to oxidative stress (136). Recent studies have shown that mitochondrial dysfunction plays vital role in hyperglycemic-induced neuronal damage (127, 136, 137). Oxidative stress is one of the leading causes of mitochondrial dysfunction (119). Oxidative stress can interrupt one or more mitochondrial functions, increasing membrane permeability (138). Furthermore, oxidative stress can increase neurotoxic glutamate levels, affecting mitochondrial phagocytosis (119, 139).Microglia are one of the important cells involved in neuroinflammation. They play an important role in maintaining neuronal homeostasis, neuron growth, building extra synapses, removing fragments of cells, removing protein aggregates and neuroplasticity (119, 140). Microglia can identify pathogens, protein aggregates, or fragments through pattern recognition receptors (PRRs) in pathogen-associated molecular patterns (PAMPs) and damage-associated molecular patterns (DAMP), and activate phagocytosis pathogens, release cytokines, chemokines, ROS/RNS until an immune response is eliminated (141). Due to mitochondrial dysfunction, mitochondrial membrane damage releases DAMP, which initiates multiple inflammatory cascades leading to neuroinflammation. The DAMP released by mitochondria (TLR, TNF receptor, inflammasome) can be identified by PRRs of microglia, activate the TLR/NF-κ B inflammatory pathway, and promote the release of pro-inflammatory factor cytokines and chemokines (119). Inflammation caused by the DAMP released by mitochondria can lead to mitochondrial dysfunction, increase ROS, and exacerbate inflammatory circulation (119).

Neuroinflammation is closely associated with microglia hyperactivation. Studies have shown that NLRP3/IL-1β signaling may underlie the correlation between visceral obesity and cognitive impairment in humans, with high-fat diets feeding WT and NLRP3-KO mice, WT mice activating IL1R1 signaling in microglia, leading to hippocampal IL1β accumulation and neuroinflammation, and consequently cognitive impairment, while NLRP3-KO mice are protective against obesity-induced peripheral inflammation (142). Study indicated that neuroinflammation in diabetic cognitive dysfunction is associated with autophagy, continuous hyperglycemia under diabetes can trigger activation of the NF-κ B pathway and release of pro-inflammatory factors, leading to the inflammatory response, and neuronal damage (143). Pharmacological administration of mTOR inhibitors and autophagy stimulators improves inflammation *in vivo* by inhibiting NF-κ B signaling (144). In a study of Cui, they found melatonin (MLT) could improve learning and memory in diabetes-associated cognitive dysfunction mice by activating autophagy *via* the TLR4/Akt/mTOR pathway, thereby inhibiting neuroinflammation and microglial apoptosis (145). A study showed that lncRNA MEG3 overexpression significantly improved diabetic cognitive impairments by regulating the Rac1/ROS axis, and by inhibiting mitochondria-related apoptosis. In addition, MEG3 overexpression or Rac1 inhibition promoted FUNDC1 dephosphorylation and inhibited oxidative stress and neuroinflammation (146).

### 3.3 Oxidative stress and lipid metabolism in diabetic cognitive dysfunction

Lipids are a class of organic compounds that act as structural components of cell membranes, chemical energy sources and cell signaling molecules, involving many biological processes (147). Lipids can be divided into the following categories according to their structure: fatty acids, triglycerides, sphingolipids, phospholipids, glycolipids, sterol lipids, isopropylene enols and polyketides (148). Lipid metabolism can be defined as the synthesis, storage and breakdown of lipids (149). These processes are necessary to maintain complex homeostasis and lipid diversity and produce products involved in multiple cellular processes. The liver and adipose tissue play a key role in lipid metabolism. The liver helps digest, uptake, storage, and biosynthesis of dietary lipids, which can be exported as lipoproteins to provide energy or structural components (139). Adipose tissue can be used for long-term energy storage (150). Adipose tissue can be used for long-term energy storage (150). Insulin regulates fat storage by inhibiting or stimulating fat mobilization (151). Moreover, most body cells can synthesize cholesterol, but the amount depends on the cell’s needs (152).

Lipids are abundant in the brain, accounting for about 50–60% of the dry weight, especially fatty acids, glycerophospholipids, sphingolipids, and cholesterol (153, 154). A study has shown that “adipose inclusions” or “lipid particles” can be found in AD brains (155). In neurons, oligomeric *Aβ* peptides can alter cellular cholesterol metabolism (156). Obesity and abnormal blood lipids are the main risk factors for cognitive dysfunction in diabetes mellitus (157). Physiological studies have found that cholesterol metabolism, inflammation, and innate immunity are closely related to neurodegenerative lesions (158). Previous studies have found that several risk factors for Alzheimer’s disease (AD) involve genes for lipid metabolism and transport, such as *APOϵ4*, *CLU*, and *ABCA7*(159–161). Therefore, abnormal lipid metabolism may be important in diabetes-associated cognitive dysfunction.

Lipid types and levels in the brain are vital determinants of brain function. Studies found that human liposomes change with age and aging can cause damage to the distribution of brain lipids and cause brain dysfunction (162, 163). The increase in oxidative stress is one of the signs of aging. Redox imbalance in the body damages to the cellular mechanism (164). Increased levels of ROS and RNS in patients with DM and AD (48, 165). Significant increase in oxidized proteins and lipids in patients with the brain in AD (166, 167). Studies such as Cutler found that changes in sphingolipid and cholesterol metabolism caused by membrane-related oxidative stress can cause neurodegenerative cascades (168). Increased oxidative stress and lipid peroxidation are associated with cognitive dysfunction in diabetes mellitus. One of the most significant hypotheses for neurodegenerative lesions is the amyloid hypothesis, which holds that excessive amounts of insoluble A subtypes cause tau to be over phosphorylated, resulting in free radical generation, inflammation, and oxidative damage (169). Low-density lipid lipoprotein receptor-related protein (LRP1) is involved in the clearance of Aβ peptide. Oxidation of LRP1 will inhibit its ability to remove Aβ peptide, resulting in the accumulation of Aβ peptide in the brain (170). High concentrations of ROS can lead to increased lipid peroxidation in the brain and change membrane permeability and membrane receptor and associated enzyme activity (171). Lipid peroxidation produces active aldehydes, such as malonaldehyde (MDA) and 4-hydroxynonenaldehyde (4-HNE), which combine and modify proteins involved in metabolism, antioxidant defense systems, and axon growth. The tau protein can be modified by 4-HNE, which indirectly leads to increased entanglement of neuronal fibers (172). In addition, LRP1 is also a covalently modified protein that further leads to the production of lipid peroxidation products. These products can cause normal initiation cascade dysregulation in neurons (173).

Several studies have confirmed the presence of disorders of lipid metabolism, including SP metabolism, Trp metabolism, and GP metabolism, in both patients with cognitive impairment in diabetes or in rat models (174, 175). Lipid metabolism can regulate numerous cellular signaling pathways involved in inflammatory responses (e.g., fatty acids, diacylglycerol (DAG), sphingolipids, CD36, Lipocalin 2). Microglia lipid metabolism is specifically involved in the control of microglia activation and effector functions such as migration, phagocytosis and inflammatory signaling, and minor disturbances in microglia lipid processing are associated with altered brain function in diseases characterized by neuroinflammation (176). Furthermore, peroxides produced by lipid peroxidation interfere with the structure of the cell membrane and protein function and stimulate intracellular signaling and other pathways leading to cell death. As a type B scavenger receptor, CD36 recognizes low-density lipoprotein (LDL), oxidized phospholipids, and beta-amyloid and is also an FA transporter. By activating the innate immune response, phagocytosis, and oxidant production in microglia, cd36g orchestrates transcriptional and metabolic remodeling. By recognizing oxidized parenchymal LDL receptors (TLRs) and internalizing receptor-ligand complexes, cd36 triggers Toll-like responses to stimulate sterile inflammation, which is closely related to neuroinflammation in cognitive impairment and mild inflammation in obesity (176). Lipocalin 2 (LCN2) functions in the regulation of the immune system and inflammatory processes. LCN 2 is mainly produced in the glia of the brain under oxidative stress and can disrupt the blood-brain barrier by promoting astrocyte and brain endothelial cell damage. Studies have shown that LCN2 regulates cellular activity in the central nervous system, controlling iron accumulation, and modulating neuroinflammation by activating glial cells (177). It may regulate neuroinflammation by activating the NF-κB signaling, activating signal transduction and transcription 3 (STAT 3) pathway to activate microglia, promoting astrocyte activation, further activation of microglia, and inhibiting neuroprotective cell pathways in the brain by regulating cytokines such as IL-1β, TNF-α, and IL-6 (178). LCN 2 is associated with inflammatory responses in metabolic disorders (including obesity and insulin resistance), where the pathway involves NF-κB, C/EBP, and estrogen response elements (179, 180). Moreover, insulin induces LCN-2 expression, thought to be *via* the phosphatidylinositol 3-kinase and mitogen-activated protein kinase signaling pathways (180). Excess ROS, RNS are highly susceptible to oxidation of lipids containing carbon-carbon double bonds, especially polyunsaturated fatty acid (PUFA), peroxides of PUFA and their reactive aldehydes, their end products-reactive aldehydes such as 4-HNE-lead to protein carbonylation, and 4-HNE and other lipophiles mediate cytokinesis through protein adduct toxicity (181). Furthermore, 4-HNE processing activates pathways including DNA damage, antioxidant, ER stress, and heat shock responses associated with neuroinflammation, insulin resistance, and other diseases (182).

## 4 Conclusions and perspectives

Recent epidemiological and experimental data provide evidence of a bidirectional interaction between obesity and cognitive dysfunction in diabetes. Obesity and diabetes are both risk factors for cognitive dysfunction, and cognitive dysfunction in diabetes is one of the complications of diabetes (78). Therefore, hyperglycemia in diabetic patients is a cause of cognitive dysfunction, while obesity is the primary cause. Insulin resistance, neuroinflammation, and lipid metabolism disorders are exacerbated by obesity in diabetic patients, resulting in cognitive dysfunction in diabetic patients. The physiological processes of obesity and diabetic cognitive impairment are mediated by biological processes of oxidative stress. Oxidative stress in obese tissues is mainly caused by the disruption of the adipose microenvironment, chronic low-grade inflammation, and mitochondrial dysfunction. Furthermore, oxidative stress affects insulin resistance, neuroinflammation, and lipid metabolism in diabetic brain tissue, affecting physiological and pathological processes. In addition to accelerating the destruction of neurons, aging, and tau protein deposition in the brain, diabetic brains are prone to cognitive dysfunction. The obese diabetic population should be regularly evaluated for cognitive function because obesity is a risk factor for diabetic cognitive dysfunction. Managing diabetic cognitive dysfunction disease in obese and metabolically impaired individuals requires meticulous management, including antioxidants if necessary.

## Author contributions

HL and JR designed the work of review; HL, JR, and YL reviewed the literature available on this topic and wrote the paper; HL and JR contributed to the scientific writing of the manuscript; HL, JR, QW, and JW revised the manuscript. HL, JR, YL, QW, and JW contributed equally to this work. All authors contributed to the article and approved the submitted version.
